# Ontogeny of Polycotylid Long Bone Microanatomy and Histology

**DOI:** 10.1093/iob/oby007

**Published:** 2019-01-02

**Authors:** F R O’Keefe, P M Sander, T Wintrich, S Werning

**Affiliations:** 1 Department of Biological Sciences, Marshall University, One John Marshall Drive, Huntington, WV, USA, and Natural History Museum of Los Angeles County, 900 Exposition Boulevard, Los Angeles, CA 90007, USA; 2 Division of Paleontology, Steinmann Institute, University of Bonn, Nussallee 8, 53115 Bonn, Germany, and Dinosaur Institute, Natural History Museum of Los Angeles County, 900 Exposition Boulevard, Los Angeles, CA 90007, USA; 3 Department of Medical and Health Sciences, Des Moines University, 3200 Grand Avenue, Des Moines, IA 50312, USA

## Abstract

Plesiosauria is an extinct clade of diapsid marine reptiles that evolved in the Late Triassic and radiated globally for the remainder of the Mesozoic. The recent description of a pregnant specimen of *Polycotylus latipinnis* demonstrates that some plesiosaurs were viviparous. To establish a baseline of histological data on plesiosaur ontogeny, we sampled the mother and fetus of the gravid plesiosaur specimen. To widen the base of data concerning ontogeny and life history of plesiosaurs, we gathered additional morphologic and histologic data from a securely identified growth series of polycotylids from the Pierre Shale of South Dakota. Paleohistological thin sections were prepared from the three humeri. Both adults show a dense, heavily remodeled cortex consisting entirely of longitudinally oriented secondary osteons, except for a thin rind of superficial primary bone. The mother exhibits an external fundamental system, indicating it was fully mature; the other adult does not. In both adults the cortex grades into a spongy medulla, comprising large vascular canals and erosion rooms surrounded by secondary lamellar trabecular bone, and lacking a marrow cavity. The fetal humerus possesses a medullary region similar to that of the *Dolichorhynchops bonneri* adult, although its lamellar bone is primary and deposited around calcified cartilage. The medulla is demarcated from the cortex by a prominent Kastschenko’s line. The cortex of the fetus is a relatively thin layer of periosteal woven bone, longitudinally to radially vascularized, and interfingered with columns of osteoblasts surrounded by rapidly-deposited extracellular matrix. The neonate humerus resembles the fetus, with its trabeculae identical in both size and histology, although it lacks calcified cartilage. The cortex is also similar but much thicker, consisting entirely of rapidly deposited, radially vascularized, woven to fibrolamellar bone. The cortex carries a line near its surface. This feature is not a line of arrested growth, but a sudden change in vascular angle and increase in bone density. We argue this feature is a birth line indicating a change in growth regime, possibly in response to increased hydrodynamic forces after birth. The birth line indicates that the neonate was about 40% of maternal length when born. Our histological data demonstrate that polycotylids had very high fetal growth rates, and that birth size was large. Comparison with the geologically oldest plesiosaur confirms that rapid growth evolved in the Triassic, although histological details differ, and the degree to which the polycotylid ontogenetic pattern is generalizable to other plesiosaurs is currently unknown. Further histological research utilizing full growth series is needed, particularly for Jurassic taxa.

## Introduction

**Fig. 1:**
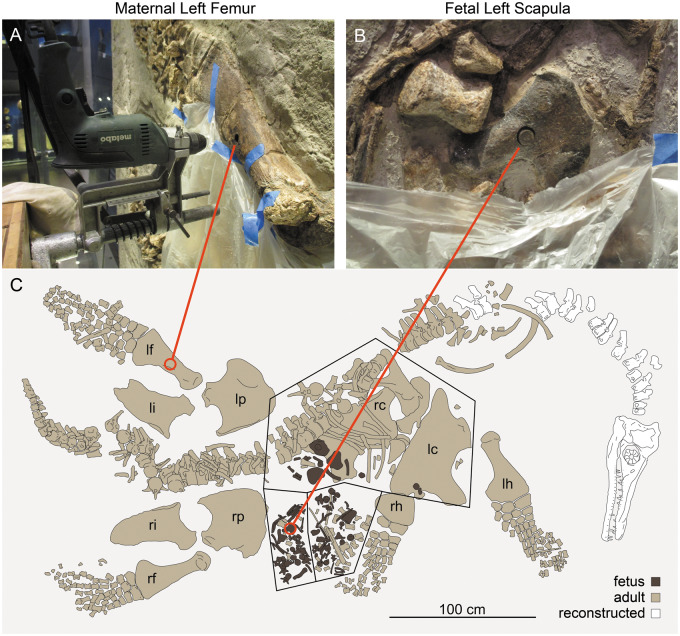
Histological sampling of *P. latipinnus*, LACM 129639. (**A**) shows jig and core drill in position for sampling of the maternal left femur. (**B**) depicts sampled core in place within the fetal left scapula; (**C**) is a schematic of the mount as displayed at the Natural History Museum of Los Angeles County (LACM). lc, left coracoid; lf, left femur; lh, left humerus; li, left ilium; lp, left pubis; rc, right coracoid; rf, right femur; rh, right humerus; ri, right ilium; rp, right pubis.

Plesiosauria was a diverse and long-lived clade of Mesozoic marine reptiles, evolving from within clade Eosauropterygia in the Late Triassic (Benson et al. 2012; Wintrich et al. 2017) and radiating rapidly through both the Jurassic and Cretaceous before meeting their demise in the K/Pg mass extinction (O’Keefe 2001a; Benson and Druckenmiller 2014). Plesiosaurs are thought to have been viviparous, based on the description of a gravid adult of the polycotylid plesiosaur *Polycotylus latipinnus*, now on display at the Los Angeles County Museum of Natural History (LACM) (Fig. 1; O’Keefe and Chiappe 2011). In this article, we test several inferences from that study, including that plesiosaurs gave birth to live young, and that the offspring were physically large when born. These hypotheses are the primary conclusions of O’Keefe and Chiappe (2011); the fetal material is certainly from a single individual, and is large, estimated at 28% of maternal length at the time of death. O’Keefe and Chiappe (2011) extrapolated from ontogenetic criteria in the fetus to predict a birth length of 40% maternal length. This size is much larger than other marine reptiles, and equaled in few extant sauropsid taxa such as the social Solomon Islands and shingleback skinks. By analogy with these taxa, O’Keefe and Chiappe predicted that *Polycotylus* may have exhibited maternal care and other social behaviors. However, the LACM specimen is one fossil, and other hypotheses are possible to explain the co-occurrence, such as intrataxic predation or chance preservation. Fortunately, such hypotheses are open to test with data from other sources. Here we employ paleohistological methods to gather data on plesiosaur ontogeny from the LACM mother and fetus, and then use these data to interpret a histological growth series from a closely related taxon. Our findings are consistent with the claim of viviparity in polycotylids, and demonstrate that birth size in the plesiosaur taxa *Polycotylus* and *Dolichorhynchopsbonneri* was very large, almost 40% of maternal length, as predicted in O’Keefe and Chiappe (2011).

### Background

The study of bone microstructure in fossil vertebrates has a long history (Padian and Lamm 2013), rooted in the classical histological and morphological traditions of the 19th and early 20th centuries. However, several factors impact bone formation and the tissues it produces, including ontogeny, physiology, biomechanics, and phylogeny, and teasing apart these factors is not easy. The 1980 de Ricqlès' publication on dinosaur bone histology was a pivotal contribution to the evolving debate on dinosaur physiology (de Ricqlès 1980), spawning both controversy and an entire field of inquiry (Chinsamy 1994; Horner et al. 1999, 2000; Erickson and Tumanova 2000). Histology was seen to be a powerful source of data about the growth and physiology of extinct animals, but also carried biomechanic and taxonomic signals, requiring care in interpretation (Cubo et al. 2008; Montes et al. 2010; Huttenlocker et al. 2013; Padian and Lamm 2013). Despite these challenges paleohistology has become a vital tool in 21st-century paleontology, rendering deep insights into end Permian extinction dynamics (Botha-Brink et al. 2016), the dwarfing of sauropod dinosaurs (Sander et al. 2006; Stein et al. 2010), the growth of hatchling sauropod dinosaurs (Curry-Rogers et al. 2016), and biomechanic convergence and diversity in secondarily aquatic tetrapods (Houssaye et al. 2016).

The change in biomechanic regime implicit in the return to water exerts a powerful influence on bone anatomy, microanatomy, and histology in many clades, but this impact is modulated by phylogeny, physiology, and above all by the degree of secondary aquatic adaptation in individual taxa (Houssaye et al. 2016). Bones also vary markedly within a single organism; axial microanatomy and histology are quite different from those of the appendicular in many clades. Axial bone microanatomy is governed by buoyancy regulation, and pachyostosis evolves repeatedly, but the overall trend is toward light, osteoporotic vertebrae and ribs in pelagic animals as the demands of support against gravity are relaxed (Houssaye et al. 2016; Krahl et al. 2013). Adult plesiosaurs are similar to ichthyosaurs, cetaceans, and derived mosasaurs in exhibiting lightening of the vertebrae relative to terrestrial taxa (Wiffen et al. 1995). The ribs resemble those of some mosasauroids and primitive cetaceans in being slightly osteosclerotic with a spongious medullary cavity comprising thick trabeculae (Houssaye et al. 2016). Pachyostosis is relatively rare in plesiosaurs but does occur, notably in the sirenian-like pliosaur *Pachycostasaurus* (Cruickshank et al. 1996). Axial pachyostosis is significant but limited to the gastralia in the cryptoclidid *Tatenectes* (Street and O’Keefe 2010), and also occurs to a modest degree in the gastralia of *Cryptoclidus* (Andrews 1910). However, these are exceptions, and in general plesiosaur axial histology is broadly similar to that of other pelagic marine tetrapods.

The appendicular skeleton loses its support function upon return to the sea, but is still heavily involved in both axial and appendicular locomotion (Storrs 1993). Limbs of aquatic taxa tend to be relatively short and robust at the gross anatomical level, with much thicker cortical bone and little or no true medullary cavity (Houssaye et al. 2016). Within Sauropterygia, plesiosaurs certainly fit this pattern; both humerus and femur are very short and robust relative to terrestrial taxa, and the distal remainders of both limbs are completely remodeled as hydrofoils (Storrs 1993; O’Keefe 2001b). The fossil record of this transition is excellent. Nothosaur-grade sauropteryians radiated throughout the Middle and Late Triassic and showed a wide range of aquatic adaptation (Klein et al. 2016). The discovery of gravid pachypleurosaurs (Cheng et al. 2004) demonstrates that these least derived of nothosaur-grade taxa were already viviparous, although plesiomorphic in retaining large brood size (Sander 1988; O’Keefe and Chiappe 2011). These small pachypleurosaurs differ little from their terrestrial antecedents, while successive clades of true nothosaurs become more adapted to life in the water (Storrs 1993; Rieppel 2000). Most of the true nothosaurs were probably amphibious, although the largest show radical bone tissue reorganization that suggests they may have been pelagic (Klein et al. 2016). The sauropterygian clade Pistosauroidea also arose in the Triassic. This clade contains Plesiosauria, as well as a poorly-resolved group of increasingly pelagic sister taxa. A gap in the record through the Norian is followed in the Rhaetian by the first known plesiosaur (Wintrich et al. 2017). A recent study of nothosaur life history has shown that whilst pachypleurosaurs and *Simosaurus* grew at rates comparable to a range of extant reptiles, their birth size was large, and they reached sexual maturity at a relatively young age (Klein and Griebeler 2018). However birth mode is not known in most taxa; within Sauropterygia, only gravid pachypleurosaurs and plesiosaurs have been described.

The propodials in pistosaurs are relatively short and robust, with delay in ossification at the ends. These trends are continued to an extreme in the first plesiosaurs (reviewed in Krahl et al. 2013; Wintrich et al. 2017). Plesiosaurs were fully pelagic, and the plesiosaur propodial remains a short and massive element throughout the subsequent history of the clade. The internal anatomy of the plesiosaur propodial was first figured by Lydekker in 1888 and has been well-characterized since then (reviewed in Liebe and Hurum 2012). The ends of the bone consist of two cones of endochondral bone whose bases form articular surfaces (Moodie 1911). The apices of these cones converge toward a small open medullary area near midshaft that receives a large nutrient artery (Moodie 1908). This space is not medullary but vascular, and splits the proximal and distal ends of the embryonic endosteal cartilage in two. The resulting hourglass shape is wrapped in a layer of periosteally-deposited cortical bone that is very thick at midshaft but narrows rapidly towards the ends. The endosteal cones are demarcated from the periosteal cuff by a prominent Kastschenko’s line (more properly Kastschenko's surface in three dimensions; Klein and Griebeler 2018), and the two often break apart, particularly in young animals (Moodie 1916; Wiffen et al. 1995). This line is obliterated by trabecular remodeling in at least some adult plesiosaur taxa (Wintrich et al. 2017; see below). The thick periosteal cuff of the plesiosaur propodial is an attractive place to look for histological information on bone deposition. The propodial cortex is a rich source of growth data because periosteal bone bears a record of its deposition rate. This principle was first articulated by Amprino (1947), and it now has a quantitative underpinning (de Margerie et al. 2002; 2004; Fleischle et al. 2018).

### Sauropterygian cortical microanatomy and histology

The bone tissue architecture of Triassic eosauropterygians has been the subject of much recent study, reviewed in Klein et al. (2016) and Klein and Griebeler (2018). Bone microanatomy and histology in nothosaur-grade taxa are surprisingly diverse although some trends are apparent. Periosteally-deposited bone is thick in the diaphysis of both humerus and femur relative to terrestrial taxa, and the medullary cavity is spongious in some taxa and open in others. Nothosaurs of large body size show radical lightening of the propodials achieved by resorption of periosteal bone. Periosteal vascularization is radial in most taxa, indicating relatively rapid growth (De Margerie et al. 2004; Fleischle et al. 2018); however, osteons are primary, and the bone fabric is generally lamellar zonal bone. Fibrolamellar bone is rare, and Haversian remodeling completely absent, in nothosaur-grade taxa (Klein 2010; Krahl et al. 2013; Klein and Griebeler 2018). These observations have been used to infer that most Triassic sauropterygians lacked an elevated body temperature, although there is evidence for modest increase in the more derived members of several nothosaur clades (Fleischle et al. 2018).

Microanatomic and histologic changes thought to indicate a major increase in body temperature are first observed in *Pistosaurus*, a Middle Triassic pistosauroid close to the origin of Plesiosauria (Fabbri et al. 2014; Wintrich et al. 2017). The femur of *Pistosaurus* resembles that of more basal nothosaurs, comprising a thick periosteal cortex enclosing a marrow cavity that is partly filled with spongy trabeculae (Krahl et al. 2013). Cortex vascularization is radial, and histology is primary lamellar zonal bone exhibiting lines of arrested growth (LAGs). Surprisingly, the humerus is quite different. There is no marrow cavity, periosteal vascularity is radial, and the bone fabric is fibrolamellar. There is no secondary remodeling, and LAGs are also present. This is the first appearance of fibrolamellar bone within pistosaurs, a bone type characteristic of plesiosaurs. The appearance of fibrolamellar bone in the humerus of *Pistosaurus* has been linked to the evolution of forelimb-driven locomotion, an increase in basal metabolic rate, or both (Krahl et al. 2013). Those authors further hypothesize that increased metabolic rate in pistosaurs was an exaptation allowing the adoption of a fully pelagic lifestyle, releasing sauropterygians from biogeographic confinement in the warm Tethys Sea and allowing the trans-Pacific spread of derived pistosaurs (Rieppel et al. 2002).

Investigation of plesiosaur microanatomy and histology has a long history, beginning with the work of Kiprijanoff in 1883, extending through elasmosaur juvenile and adult histology (Wiffen et al. 1995), and recently augmented by analyses of the stratigraphically oldest plesiosaur, by Wintrich et al. (2017). The microanatomy and histology of plesiosaur propodials—both humerus and femur—change little from the latest Triassic to the end Cretaceous. Plesiosaurs are osteosclerotic with a closed, spongy medulla, and are unique among sauropterygians in showing full Haversian remodeling of the periosteal cortex in mature adults of some taxa (Wiffen et al. 1995; Krahl et al. 2013; Wintrich et al. 2017). The presence of Haversian remodeling may suggest a high metabolic rate, in accord with isotopic data that demonstrate that plesiosaurs were homeothermic to some degree (Bernard et al. 2010). Recent quantitative analysis of plesiosaur cortical growth rates demonstrates they were very high, on a par with modern birds, and that an elevated body temperature was probably necessary to produce them (Fleischle et al. 2018). All plesiosaurs retaining primary cortex show radial vascularity with primary osteons of fibrolamellar bone. In some Jurassic pliosaurs and Cretaceous elasmosaurs the cortex is completely remodeled, replaced by secondary Haversian osteons with longitudinal vascularity. However, the Early Jurassic plesiosaur *Plesiosaurus* shows several growth lines in a fabric of primary fibro-lamellar bone, and this is the general pattern in Jurassic taxa except for true pliosaurs (Kiprijanoff 1882; Wintrich et al. 2017). However, the contributions of phylogeny and biomechanics to these differences is currently unknown. There is currently no consensus regarding the complex interplay between thermal physiology and biomechanics on bone growth, and most of the extant literature is on mammals (Currey et al. 2017). Generally, bone microanatomy and histology clearly indicate that plesiosaurs were very fast-growing, and while variation exists, this rapid growth was characteristic of all known taxa (Wintrich et al. 2017; Fleischle et al. 2018). Biomechanic forces probably also contributed to patterns of bone remodeling, but this subject has received little study in plesiosaurs.

The modern taxa whose humeri are most similar in microanatomy to those of plesiosaurs are the leatherback turtle (Liebe and Hurum 2012) and penguins. Both modern taxa are homeotherms. Penguin humerus microanatomy and histology resembles that in pistosaurs, where the forelimb is adapted to aquatic locomotion while the femur is more plesiomorphic, being pachyosteosclerotic with an open marrow cavity (Ksepka et al. 2015). Penguins fly underwater with the forelimb and walk on land with the hindlimb, and this division of function is mirrored in the bone tissues of humerus and femur, lending credence to the idea that underwater flight evolved first in the forelimb in pistosaurs (Krahl et al. 2013). Hindlimb flight evolved later, and is unique to plesiosaurs (O’Keefe and Carrano 2005). The microanatomic and histologic similarity between the humerus and femur of plesiosaurs implies strongly they were used in similar ways, presumably in underwater flight. While gait certainly varied among plesiosaurs (Long et al. 2006), all four limbs were probably involved in most cases, as modeled by Muscutt et al. (2017).

The identity, or identities, of growth marks found in the plesiosaur cortex has not been determined conclusively (see discussion in Wintrich et al. 2017). All plesiosaurs that preserve a propodial growth record show a very rapid first growth phase, characterized by radial vascularity and woven fibered bone to fibrolamellar bone, followed by a sudden change in vascular angle, forming a first growth mark (Wintrich et al. 2017). Wintrich et al. identify this feature as a yearling growth mark in the Triassic *Rhaeticosaurus*; this is consistent with the high growth rates documented by Fleischle et al. (2018). This growth mark might also represent a birth line, but if this were true it would indicate a birth length of 60% of maternal length for the fetus (Wintrich et al. 2017), which seems implausible. In *Rhaeticosaurus*, *Plesiosaurus*, and *Cryptoclidus*, this first growth mark is followed by additional cortical growth marks of the same kind, always characterized by a sudden reorientation of the vascular canals in the fibrolamellar bone. Growth marks laid down during the phase of substantial growth are thus very different from the more typically sauropsid LAGs and lamellar-zonal bone found in most nothosaurs and *Pistosaurus*. However, like the LAGs in non-plesiosaurian sauropterygians, the regularly spaced growth marks of plesiosaurs are presumably yearly in nature (Wintrich et al. 2017). In some plesiosaurs, closely spaced LAGs in the outermost cortex form an external fundamental system, recording the cessation of growth (Krahl et al. 2013, Fig. 11C). Lastly, the intense secondary remodeling found in many adult plesiosaur specimens obscures completely any growth record (Wiffen et al. 1995, Krahl et al. 2013; Wintrich et al. 2017). The timing of secondary remodeling in adult plesiosaurs cannot be constrained due to the lack of good growth series for most taxa.

## Materials and methods

We first sampled the adult and fetal skeletons of the gravid LACM *Polycotylus* specimen, allowing us to identify tissues that are certainly prenatal in the fetus. Unfortunately, no propodials are present in the fetal skeleton (*contra*O’Keefe and Chiappe 2011; O’Keefe and Byrd 2012), so we sampled the left scapula, which is more proximal than the humerus but still appendicular (Fig. 1). We also sampled the left femur of the mother because the specimen is on display and both humeri are near eye level, while the left femur is high on the wall and the damage less obvious. Plesiosaur propodials have very similar anatomy and histology, allowing comparison between humerus and femur (Houssaye et al. 2016; Wintrich et al. 2017).

The second set of data we utilize is an ontogenetic series of three plesiosaur polycotylid humeri. This material consists of two partial skeletons originating from the Pierre Shale of South Dakota, held now at the University of Nebraska State Museum (UNSM), and an isolated humerus from the Field Museum of Natural History (FMNH). Slightly stratigraphically younger than the LACM material, the two USNM skeletons are reviewed and referred to the taxon *D.bonneri* by Morgan and O’Keefe (in press). Polycotylid ingroup relationships are notoriously unstable (O’Keefe 2008), although recent progress has been made (Fischer et al. 2018; Morgan and O’Keefe in press), and the *D. bonneri* entity is certainly closely related to *Polycotylus*. The two UNSM skeletons vary greatly in ontogenetic age, with one a full adult, and the other a young juvenile based on ossification state of the skull and vertebrae (Morgan and O’Keefe in press). The FMNH propodial is a small isolated polycotylid humerus. Little is known about this humerus except that it also comes from the Pierre Shale, it is certainly a polycotylid, and is ontogenetically very immature.

Together these three humeri yield a feasible growth series of a large polycotylid. By using propodial length as a size proxy, we can pose a series of hypotheses about microanatomy and histology and then test them with reference to the known fetal and adult tissues from the LACM material. Based on gross anatomical examination and measurement (see section “Results”, Table 1), we are able to pose several hypotheses concerning size and ontogenetic age in the *D. bonneri* material. The size estimate used here is propodial length, and we are therefore assuming that propodial lengths scale isometrically with body size. While this is known to be untrue (Kear 2007), the documented allometries are modest compared to the scale of the size differences in the growth series. The first hypothesis is that the large *D. bonneri* skeleton is an adult, because it is larger than the LACM mother (106%) and shares other adult features (see Results below). We therefore hypothesize that the large *D. bonneri* humerus will show adult histological features, with complete Haversian remodeling. The second, immature *D. bonneri* skeleton is about 40% the size of the adult. We hypothesize it is a neonate, because this is near the birth size predicted by O’Keefe and Chiappe (2011). The bone tissue should be largely fetal, although there may be birth lines or the onset of remodeling. Lastly, because the isolated humerus is 28% the length of the adult, we predict it comes from a fetus. It should display fetal bone tissue only. Another possibility is that the isolated humerus is from a much smaller taxon than *D. bonneri*; if this is the case we should see more mature bone tissue than the size of the element would suggest.

**Table 1:** Length measurements for humeri and femora histologically sampled in this paper

Specimen	Age	Taxon	Humerus L, mm	Femur L, mm	Adult length, %
LACM 129639m	Mother	*P. latipinnus*	476	501	100
LACM 129639f	Fetus	*P. latipinnus*	*	*	32 (based on other elements)
FMNH PR 1619	Small	Polycotylidae	150	*	29
	Juvenile				
UNSM 55810	Juvenile	*D. bonneri*	208	*	40
UNSM 50133	Adult	*D. bonneri*	516	516	106

### LACM histological sampling

Samples were obtained from the left femur of the mother (LACM 129639a) and the left scapula of the fetus (LACM 129639b; Fig. 1). In the adult, the left femur is most suitable for sampling because the other stylopodial bones are incomplete (right humerus, right femur) or poorly preserved in the shaft region (Chiappe and O’Keefe 2011). In the fetus, the scapulae are well preserved and accessible. The two bones were sampled for histology by coring at the midshaft of the ventral diaphysis of the maternal femur, and the base of the ventral aspect of the fetal glenoid ramus of the scapula. The maternal femur was sampled using a 18 mm core bit, and the fetal scapula was sampled using a 11 mm bit. The choice of sample location on the femur was based on comparison with other plesiosaurs (see Wintrich et al. 2017) where the location of the medullary cavity is indicated by the nutrient canal, visible in the left femur on its posterior margin. The femur core sample reached the medullary region at a depth of about 40 mm but did not traverse it. The scapula core sample traversed the entire thickness of the bone, revealing both cortices (ventral and visceral) and the medullary region.

An added challenge to sampling is the vertical position of the LACM exhibit mount. The mount required modifications to the coring technique of Stein and Sander (2009), see also Sander (2000), to adapt it to horizontal drilling on a vertical slab. The drill press holding the electric drill was bolted to a vertical board attached to a scissors platform with C clamps, resulting in a horizontal position of the drill press (Fig. 1). Below the sample site, the specimen was covered with a plastic tarp reaching the floor and collecting the water serving as drilling coolant. All other steps of drilling and sample recovery adhered to the method described by Stein and Sander (2009).

The cores of the mother and the fetus were embedded in epoxy resin and sectioned along their long axis in a plane perpendicular to the long axis of the bone (Stein and Sander 2009). The plane bisecting the core thus represents a segment of the entire cross section of the bone (Sander 2000). One half of each core was then processed into a standard petrographic thin section 50–80 μm in thickness (see Lamm 2013). The sections were observed under a Leica DM2500LP polarizing microscope, and digital photomicrographs were taken with a Leica DFC420 color camera mounted on this microscope and edited using the 2007 Leica IMAGE ACCESS EASYLAB 7 software. Overview images of the sections were obtained with an Epson V750 high resolution scanner.

### FMNH/UNSM histological sectioning

**Fig. 2:**
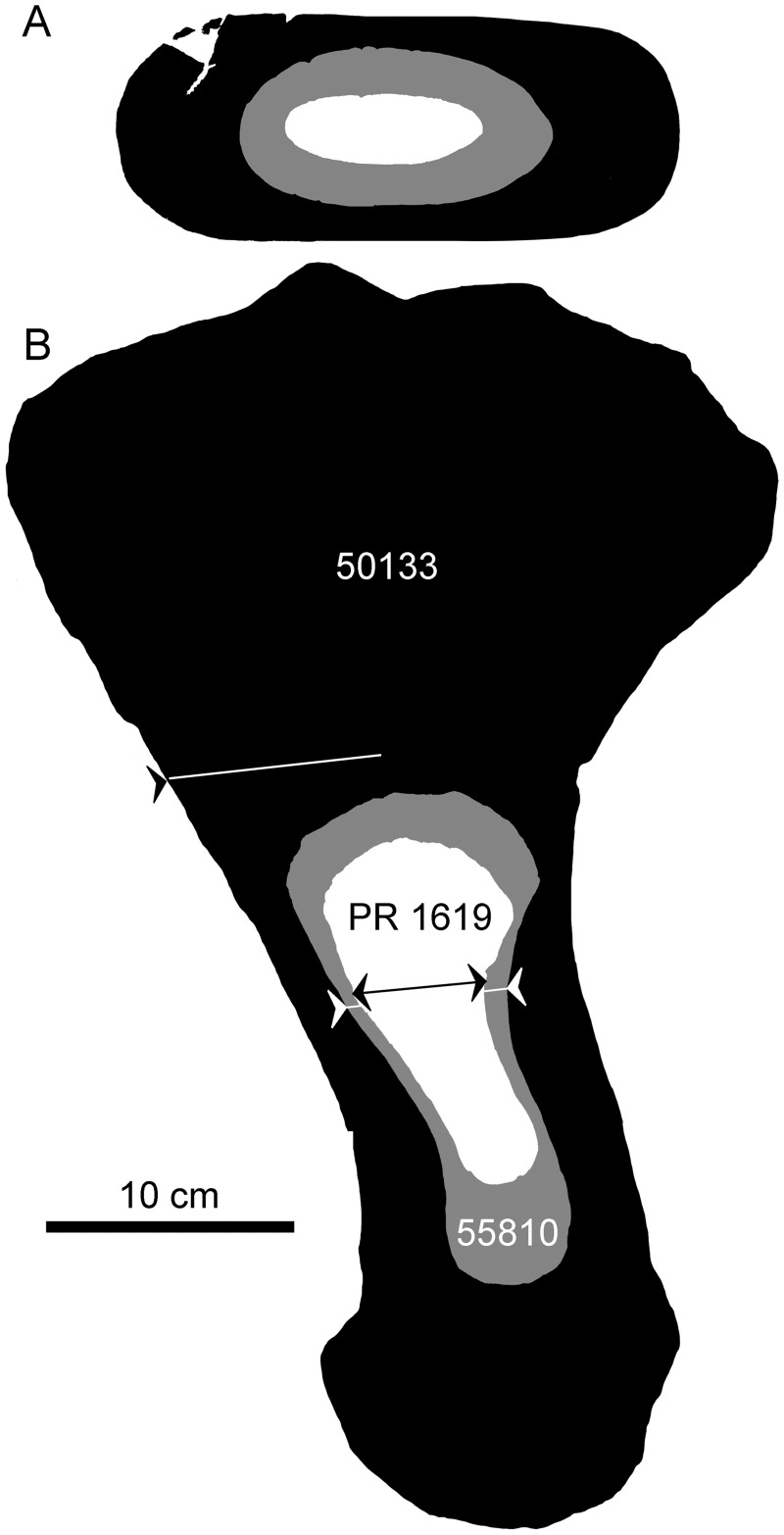
Scaled outlines for the adult, juvenile, and small juvenile humeri of the Pierre Shale material (UNSM 50133 and UNSM 55810 are referable to *D. bonneri*; FMNH PR 1619 is referable to Polycotylidae). Lines indicate the approximate planes of thin section preparation. The juvenile sections are complete, while the adult section consists of the anterior half of the shaft. For length comparisons see Table 1.

The three other histological sections described here were prepared at the FMNH. The sections are from the right humerus in all cases. The sampled right humerus of the juvenile *D. bonneri* consists of the distal end to about midshaft. The right humerus of the adult *D. bonneri* is also fragmentary, yielding a partial section of the leading edge just distal to midshaft. Before sampling, a mold was prepared of each specimen; this procedure and subsequent steps followed Lamm (2013). Each bone was mounted securely in a water-lubricated rock saw and sectioned distal to midshaft, at a location about two-thirds down each shaft (Fig. 2). This location is slightly distal to that used in the LACM adult. Sampling at this location sacrifices a small amount of the cortical growth record, but does avoid the vascular cavity at the base of the nutrient artery. This allows better characterization of the trabeculae in the endochondral cones because the tips of the cones do not touch at midshaft (Liebe and Hurum 2012). A thin wafer of bone cut from the broken ends of the two partial elements. The complete small juvenile humerus was sectioned at a similar location. All sectioned bones were then embedded in epoxy resin and secured to slides using a thin solution of cyanoacrylate. The two smaller slides were glass, while the adult was mounted on acrylic due to its size. All sections were then ground to about 80 μm thickness using a water-lubricated polishing wheel and three successively finer grades of polish. Full-slide photographs were taken using a Canon 100 mm macro lens mounted on a Canon 60D digital SLR (18.6 megapixel). Microanatomical and low resolution histology photographs were taken with the same camera body attached to a Leica S8APO dissecting scope via a phototube. High resolution histology, including under polarized light, was photographed on a dedicated histological microscope at Des Moines University.

## Results

### Anatomic description

The adult femur from the gravid LACM specimen is described in O’Keefe and Chiappe (2011). It is a typical polycotylid femur, with a long, straight shaft, a prominent trochanter positioned directly dorsal to the shaft axis, a clear periosteal division between the femoral head and the trochanter, and discrete facets for four ossifications in the epipodial row (O’Keefe 2004; Schumacher and Martin 2016; Fig. 1). The maternal femur measures 501 mm in greatest length, 25 mm longer than the maternal humerus (Table 1). As discussed above, the propodials are not known from the LACM fetus (contra O’Keefe and Chiappe 2011), and the scapula is sampled here instead; this element is described in detail in O’Keefe and Byrd (2012).

**Fig. 3:**
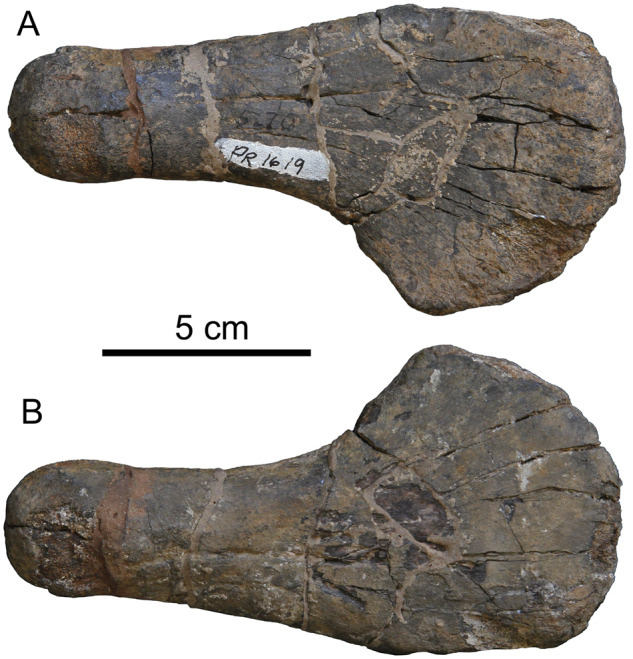
Isolated right humerus, FMNH PR 1619, referable to Polycotylidae. Top is dorsal view, bottom is ventral.

Figure 2 shows schematic, scaled outlines of the *D. bonneri* (UNSM/FMNH) growth series. The adult outline is taken from the complete left humerus, while we sectioned the fragmentary right, and the proximal part of the neonate humerus is reconstructed. Approximate planes of each histological section are indicated by lines. The isolated right humerus FMNH PR 1619 (Fig. 3) is small, measuring 15 cm long, and ontogenetically immature. The capitulum and tuberosity are poorly ossified and confluent, and the distal facets for the epipodial row are indistinct. The bone surface is cracked and poorly preserved. This is probably due to postmortem decomposition rather than recent weathering (see Results below). The element as a whole has experienced little compression. Most exposed bone is periosteally derived with only the proximal and distal faces exposing the endochondrium. These faces do not bear the large vascular foramina that are characteristic of adult plesiosaurs (Fleischle et al. 2018). However, the humerus is typically polycotylid, with an offset tuberosity, a broad distal margin, and a clear sigmoid curvature to the shaft. Surprisingly, the overall shape of this element is more similar to that of the adult than it is to the juvenile *D. bonneri* humerus.

**Fig. 4:**
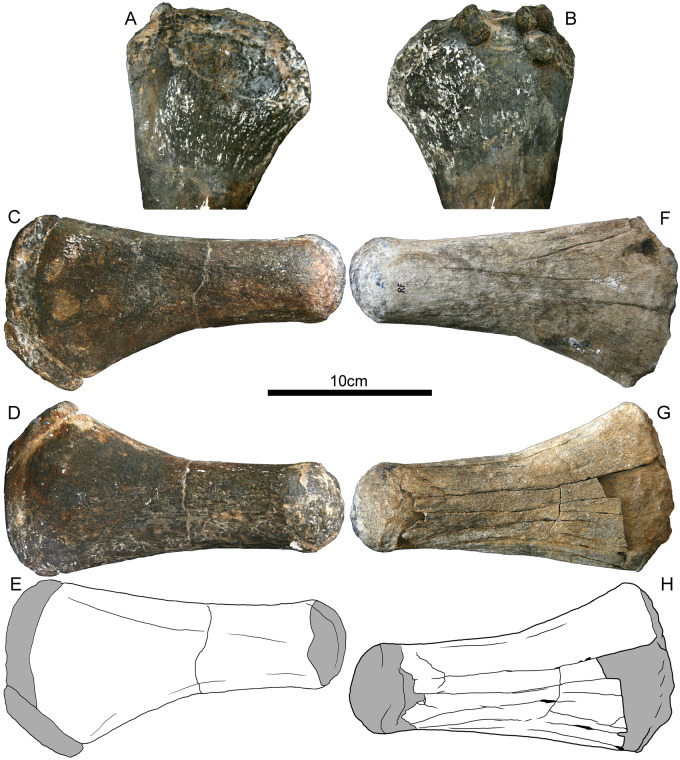
Propodials of *D. bonneri*, UNSM 55810. (**A** and **B**) show the partial, thin-sectioned right humerus; (**C** and **D**) show dorsal and ventral views of the complete left femur, (**F** and **G**) show dorsal and ventral views of the complete but weathered right femur. Drawings in (**E** and **H**) depict the periosteally-derived cortex in white, and the endochondrum in gray. Fragments of the cortex have weathered away from the endochondral cones where the cortex is thinnest, both proximally and distally.

The juvenile *D. bonneri* humerus UNSM 55180 is the distal half of the anatomical right humerus; the left humerus is missing. The skeleton also preserves both complete femora (Fig. 4) and we used the lengths of these to calculate the body size estimate in Table 1. The humerus and left femur are unweathered, but the right femur was collected on the surface and has been subjected to significant recent weathering. The distal margin of all three propodials is formed by a projection of the endosteum, extending distally from the rim of the cortical cup (Fig. 4, shown in gray). The proximal ends of the two femora are similar, with endochondral bone projecting medially past the rim of the cortex. The tuberosity and capitulum are poorly ossified and confluent, although some macroscopic vascular canals are present. The projection of endosteal bone far beyond the margin of the cortex is not present in the smaller FMNH humerus, nor is it present in the adult. None of the UNSM 55810 propodials show obvious compression. The weathering of the right femur has partially removed the cortical cones from the ventral surface of the femur, with the cortex delaminating from the endosteum along a well-defined Kastschenko’s surface. Because a complete humeral shaft is missing, we cannot establish the presence of a sigmoid curvature of the shaft, although the distal width of the humerus is polycotylid-like. The femora are also long, straight, and flared at the ends as in most other polycotylids. The femur is less taxonomically diagnostic than the humerus, although the taxonomy of this specimen is based on cranial data, and is secure (Morgan and O’Keefe in press).

**Fig. 5:**
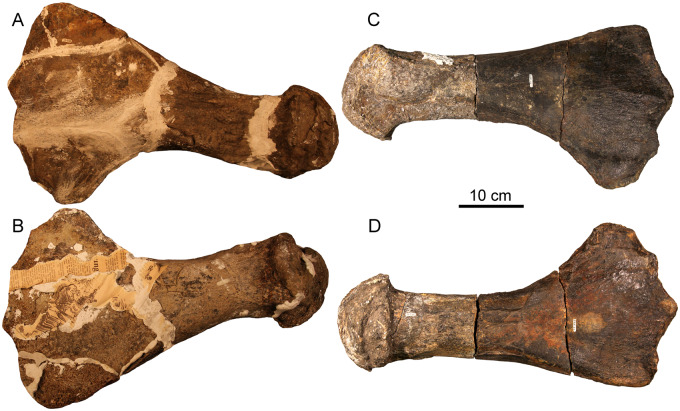
Propodials of *D. bonneri*, UNSM 50133. (**A** and **B**) show the complete left humerus in dorsal and ventral view; the complete right femur is shown in dorsal and ventral views in (**C** and **D**).

The adult *D. bonneri* skeleton UNSM 50133 includes a complete left humerus and a complete right femur (Fig. 5). These are described here; most of the left femur is also present in fragments, as is a fragment of the right humerus, which was the element sectioned. These elements are both longer than those of the gravid *Polycotylus* (Table 1). The humerus and femur are also of equal length, unlike *Polycotylus* and most other pliosauromorphs (O’Keefe and Carrano 2005). In all other respects the humerus is identical to that of *Polycotylus*, both the LACM specimen described above as well as several others (Schumacher and Martin 2016), sharing the strong sigmoid curve of the shaft, distal widening, facets for four epipodials, and offset and well-defined capitulum and tuberosity. The femur is also very similar to that of *Polycotylus* (Fig. 1); the shaft is straight and long with a broad distal expansion, and there are four well-defined facets for the epipodials. The femoral head is well-ossified and globose with a diameter significantly greater than the shaft. The trochanter is collinear with the axis of the shaft and is separated from the femoral head by cortical bone (Fig. 5). The two complete elements show evidence of brittle deformation in the dorso-ventral plane, evidenced by cracking and depression of their dorsal and ventral cortices.

### Microanatomic and histologic description

Microanatomic and histologic findings for all thin sections prepared for this article are summarized in Table 2. Our terminology follows that of Huttenlocker et al. (2013), although we retain a distinction between descriptions of vascularity and descriptions of bone fabric. Some of the fetal cortical tissues described below are initially deposited as woven bone around radial vascularity. This bone then becomes fibrolamellar with additional deposition, and some tissues are intermediate. This distinction is illustrated using polarized light to illustrate the presence or absence of birefringence, and hence the presence or absence of preferred orientation in hydroxyapatite crystals in the ground substance (Cook et al. 1962).

**Table 2:** Microanatomic and histologic summary; for further discussion see text and listed figures

Specimen	Ontogenetic Age	Taxon	Microanatomy	Cortical Histology	Trabecular Histology	Figure
LACM 129639a	Mother	*P. latipinnus*	Spongious medulla with wide voids; remodeled endosteal boundary; Pachyostotic cortex, longitudinal vascular canals. EFS present.	Secondary osteons (Haversian); some woven bone on periphery	Large erosion rooms; secondary lamellar	6
Left femur						
LACM 129639b	Fetus	*P. latipinnus*	Spongious medulla; clear endosteal boundary; periosteal cortex longitudinal to plexiform vascularity.	Primary woven fibered bone	Primary lamellar	7
Left scapula						
UNSM 50133	Adult	*D. bonneri*	Spongious medulla with wide voids; remodeled endosteal boundary; pachyostotic cortex, longitudinal vascular canals. No EFS.	Secondary osteons (Haversian)	Large erosion rooms; secondary lamellar	8,11
Right humerus						
UNSM 55810	Neonate	*D. bonneri*	Spongious medulla; clear endosteal boundary; periosteal cortex thick with longitudinal to radial vascularity; clear birth line.	Primary woven to fibrolamellar bone	Primary lamellar	9,12
Right humerus						
FMNH PR 1619	Fetus	Polycotylidae	Spongious medulla; clear endosteal boundary; periosteal cortex longitudinal to radial vascularity.	Primary woven fibered bone	Calcified cartilage; primary lamellar	10,13
Right humerus						

#### *Polycotylus* mother LACM 129639a

**Fig. 6:**
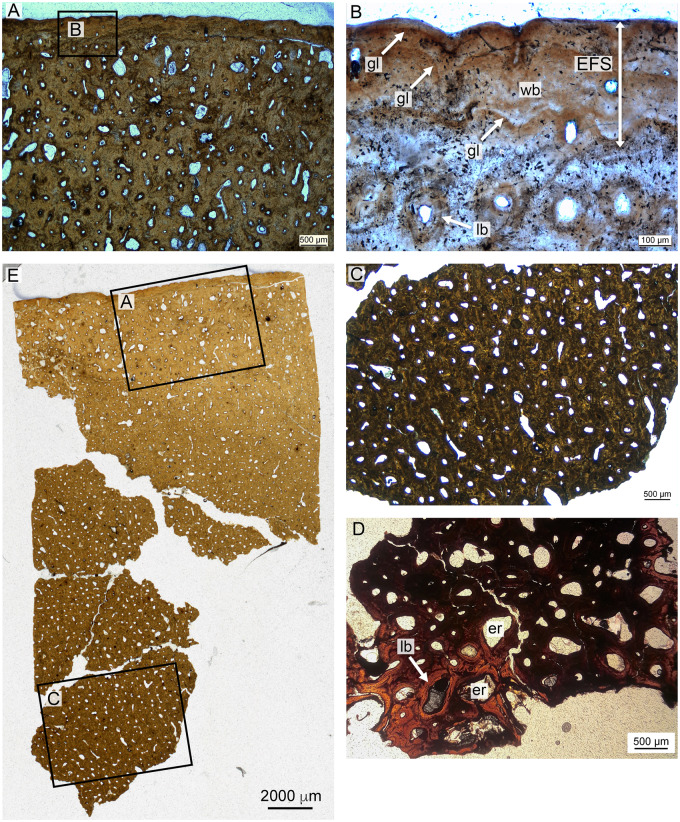
Maternal histology of the left femur, LACM 129639. (A) depicts outer cortex comprising secondary Haversian bone except for some primary woven bone near the periphery, and the External Fundamental System, magnified in (**B**). (**C**) shows secondary mid-cortical osteons, while (**D**) shows secondary lamellar bone from the medullary region. (**E**) shows the top half of the slide preparation and locations of sections A and C. Section D is from the fragmentary deep part of the core, in the cancellous trabecular region. EFS, external fundamental system; er, erosion room; gl, growth line; lb, lamellar bone; wb, woven bone.

Microscopic views of the histological preparation of the *Polycotylus* mother are presented in Fig. 6. The core sample covers a cortical thickness of about 40 mm, including a more cancellous region in the inner part of the femur. The core broke apart in the medullary region, and pieces are illustrated here. The compact bone of the cortex is almost entirely secondary, and only in the outer cortex is there any primary bone left. The outermost cortex shows a distinctive growth mark about 300 µm from the bone surface. This outermost growth cycle contains vague subcycles. The outermost growth cycle is nearly avascular, but the uneven surface of the bone with deep indentations suggests that blood vessels covered the bone surface and that the tissue may still have been actively growing, albeit slowly. The primary bone in an outer region about 8 mm in thickness is largely interstitial between secondary osteons; however, just inside the growth mark there are some primary vascular canals preserved that are oriented longitudinally. Vascularization in the secondary cortex is mainly longitudinal, but there are longitudinal secondary osteons as well, especially in a region about 20 mm from the external bone surface.

The preserved primary bone matrix in the outermost cortex is difficult to describe in terms of the classical continuum of lamellar, parallel-fibered, and fibrolamellar because it is dominated by coarse woven fibers. Many of these appear to be combined into bundles and are oriented longitudinally. The tissue thus somewhat resembles the metaplastic bone of ossified tendons (Klein et al. 2012).

In the outer region, isolated secondary osteons replace the primary bone, but from about 8 mm inward, the cortex consists exclusively of secondary osteons in multiple generations. However, the secondary osteons do not consist of normal lamellar bone matrix but also include coarse fibers. The first layer of fibers that was deposited after resorption had ceased is circumferentially oriented, while the further, centripetally deposited bone matrix consists of fiber bundles extending along the long axis of the osteon. Osteocytes are organized radially within lamellae of secondary bone tissue.

The inner part of the cortex, beginning about 30 mm from the bone surface, shows increasingly larger erosion rooms that are lined by secondary lamellar bone (Fig. 6). However, it is not clear if there was true cancellous bone. The length of the core, compared to the diameter of the bone at the drill site, indicates that the medullary region must have been small, as in other plesiosaurs.

#### *Polycotylus* fetus LACM 129639b

**Fig. 7:**
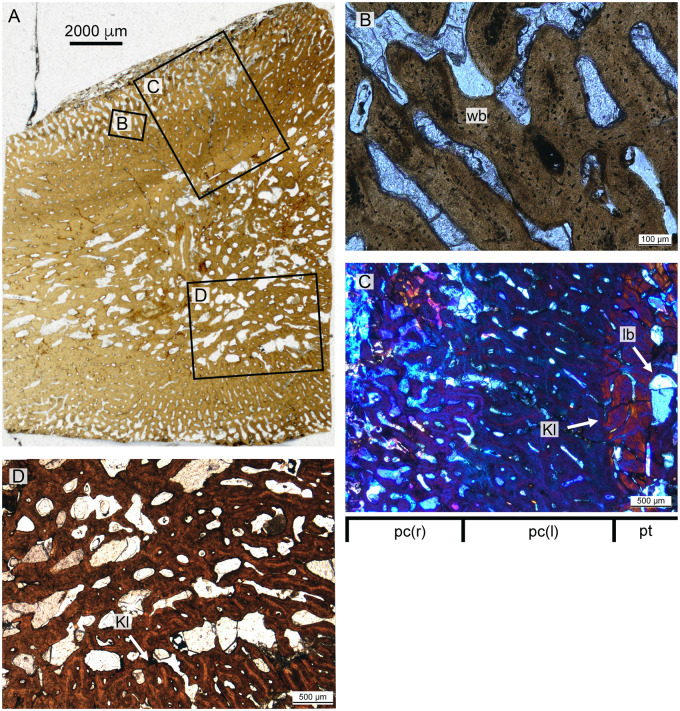
Fetal histology of the left scapula, LACM 129639. (**A**) shows an overview of the entire section, with further magnified panels indicated. (**B**) shows the mid-cortical section in detail. (**C**) in in polarized light, and indicates that the entire cortex consists of woven bone due to its lack of birefringence. (**D**) shows primary trabecular bone in the medulla, and Kestschenko's line, in normal light.Kl, Kestschenko's line; lb, lamellar bone; pr, primary radial; pl, primary longitudinal; wb, woven bone.

The core taken from the fetal scapula is shown in Fig. 7. The core passes completely through the bone, affording a view of the entire endochondrum, and both boundaries with the cortex are visible. The cortex is thin relative to the endochondrum compared to the adult humerus, although the element is a scapula and this comparison therefore suspect. The boundaries between the endochondrum and the cortex are sharp and well-defined. The cortex is heavily vascularized; this vascularity is longitudinal close to the endochondrum but becomes reticular to radial approaching the bone surface. The cortex comprises disorganized, woven fibered bone. The endochondral component is dominated by primary and secondary cancellous bone surrounding large vascular voids. The trabeculae in the cancellous bone are formed by primary lamellar bone. No calcified cartilage was identified in the endochondrum. The primary bone tissue of the fetus also shows the coarse fibrous structure seen in the mother. The secondary trabeculae running toward the posterior margin of the scapula also show coarse fibers, as in the primary periosteal bone of the mother and in the periosteal bone of other plesiosaurs.

**Fig. 8:**
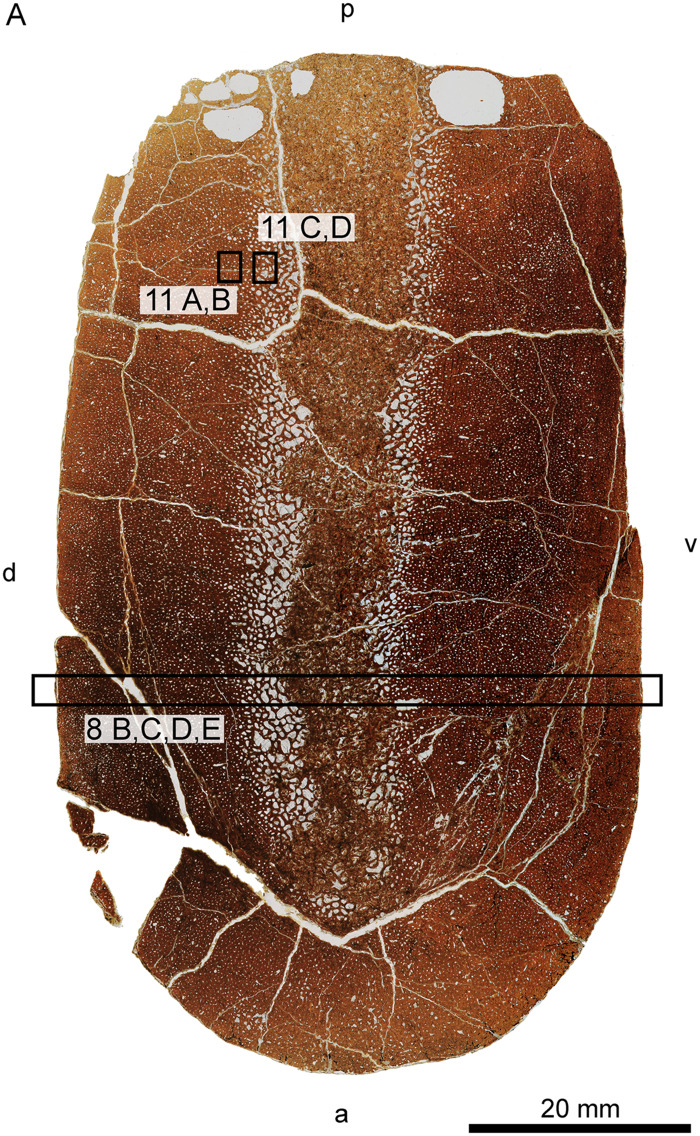

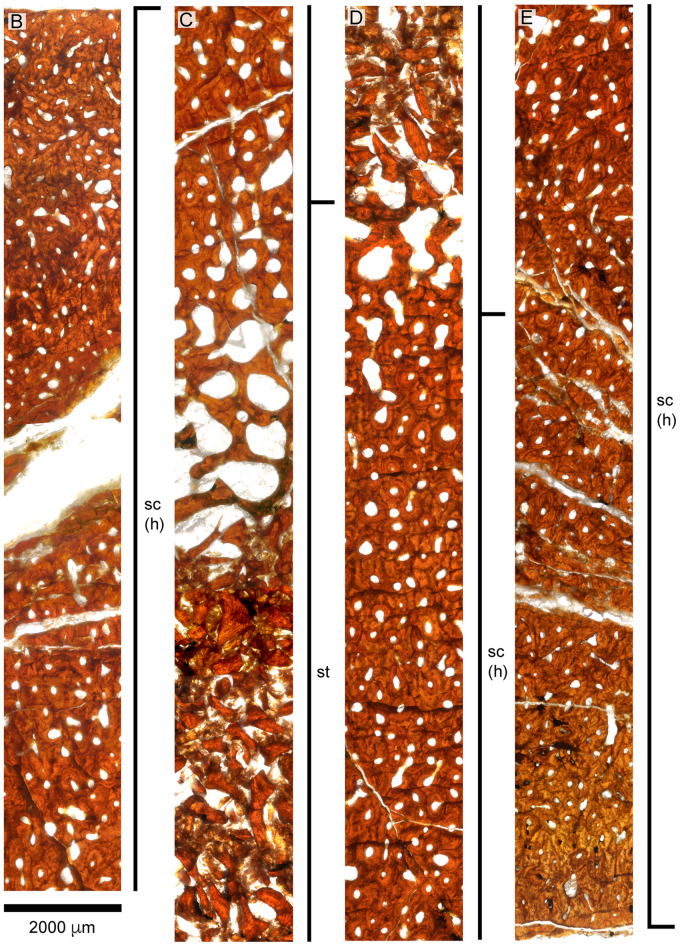
Overview (**A**) and microanatomy (**B**–**E**) of the adult *D. bonneri* humerus, UNSM 50133. Black boxes indicate magnified regions in this figure and Fig. 11. The secondary trabeculae in the center of the element have collapsed due to brittle deformation of the humerus after burial. Note lack of EFS, unlike the *Polycotylus* mother. a, anterior; d, dorsal; p, posterior; sc(h), secondary cortex, Haversian; st, secondary trabeculae; v, ventral.

#### *Dolichorhynchops bonneri* adult UNSM 50133

Histological preparation of the *D. bonneri* adult is shown in Figs. 8 and 11. The bone has a thick cortex that is completely Haversian, comprising secondary osteons with regular, longitudinal vascularity. The surface of the cortex lacks an external fundamental system (EFS), although it is possible this has been lost to weathering. This is the only microanatomic or histologic difference between this element and that of the LACM *Polycotylus.* There is no open medullary cavity; the central shaft is occupied by a spongy medulla consisting of sizable voids lined with secondary lamellar bone. The central voids in the medulla have collapsed into a jumbled mass of broken trabeculae due to brittle deformation during compression. The greater proportion of cancellous bone in this specimen compared to the LACM *Polycotylus* is due to the more distal location of the section.

#### *Dolichorhynchops bonneri* juvenile UNSM 55810

**Fig. 9:**
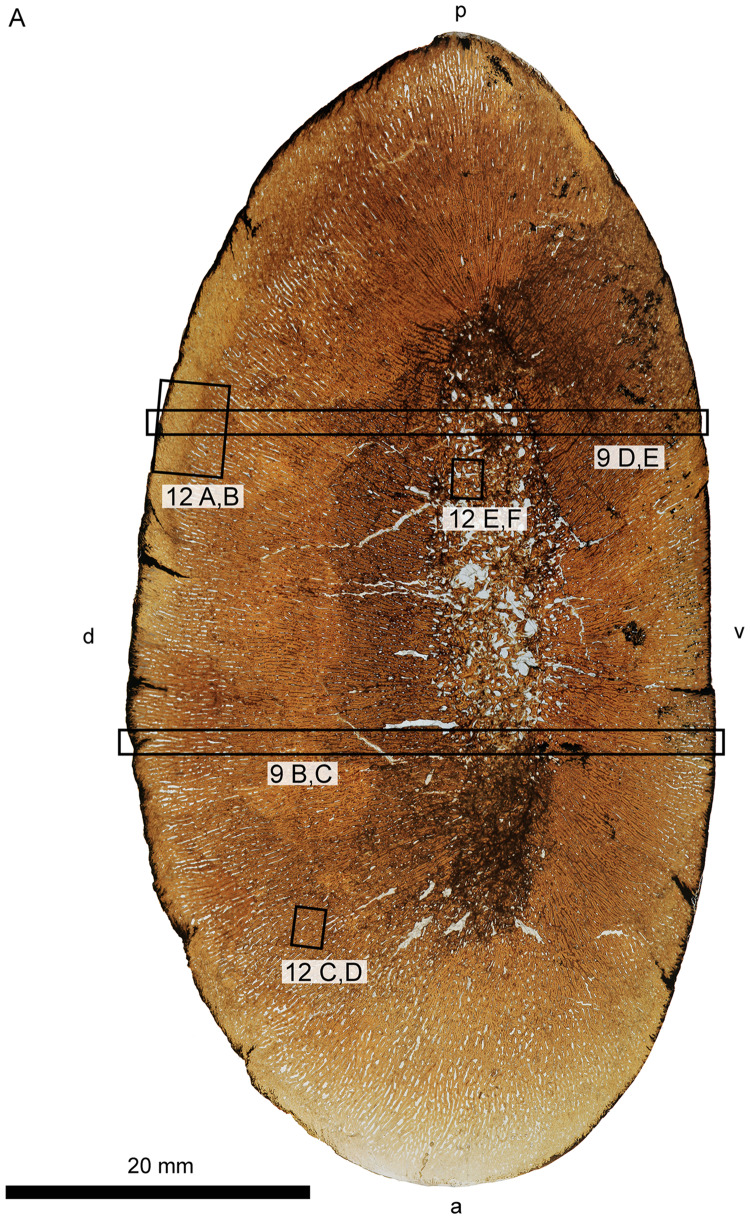

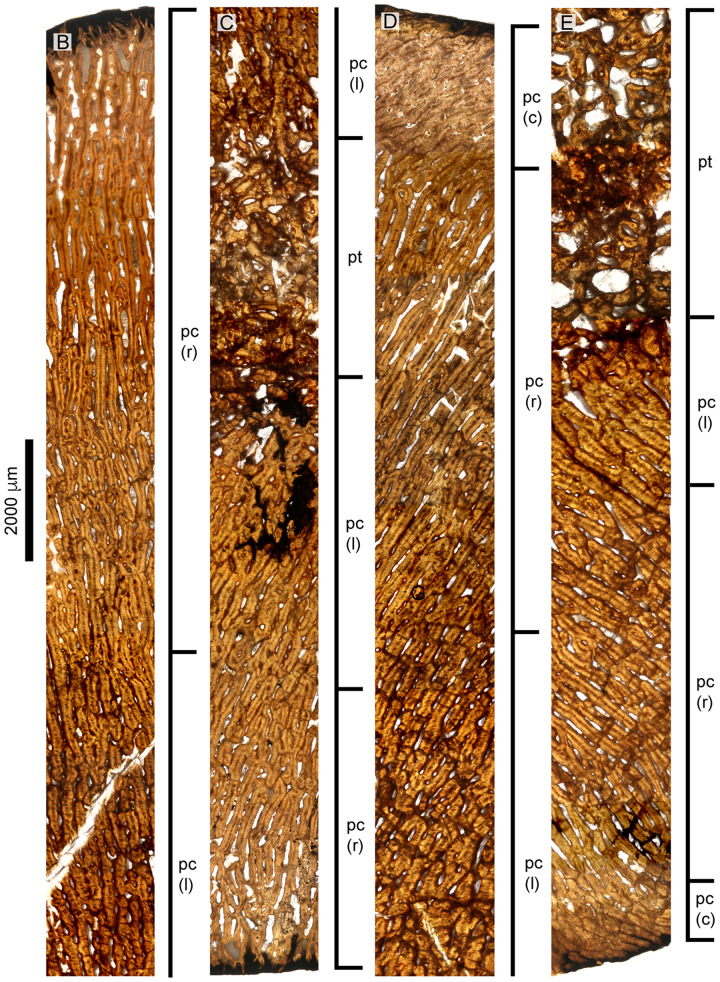
Overview (**A**) and microanatomy (**B**–**E**) of the *D. bonneri* neonatal humerus, UNSM 55810. Black boxes indicate magnified regions in this figure and Figure 12. a, anterior; d, dorsal; p, posterior; pc (r), primary cortex (radial); pc (l), primary cortex longitudinal; pc (c), primary cortex (compact); pt, primary trabeculae; v, ventral.

The histological section of the *D. bonneri* neonate humerus is presented in Figs. 9 and 12. The section is closer to midshaft than that of the adult. The bone tissue appears completely primary, with no indication of remodeling. The cortex shows longitudinal vascularity near the endosteum transitioning quickly to radial vascularity perpendicular to the long axis of the bone. Long columns of osteocytes run through the center of the bone tissue between the canals. The tissue between the vascular canals is primary woven bone close to the surface of the cortex as indicated by its lack of birefringence, but becomes pseudolamellar closer to the medulla.

A linear feature encircles the cortex for most of its diameter not far below the bone surface (Fig. 9). This feature is a change of vascular angle from radial to oblique accompanied by an increase is bone density. However the tissue here is primary coarse woven throughout, as indicated by a lack of birefringence. The linear feature is punctuated ventrally, dorsally, and posteriorly by gaps that may represent muscle attachments, although they lack obvious Sharpey’s fibers. There is no sign of an EFS. The endochondral component is demarcated from the cortex by a clear Kastschenko’s line. There is no medullary cavity but the medulla is spongy, filled with vascular voids lined with primary lamellar bone. Some of the central trabeculae have collapsed due to brittle deformation.

#### Small juvenile FMNH PR 1619

**Fig. 10:**
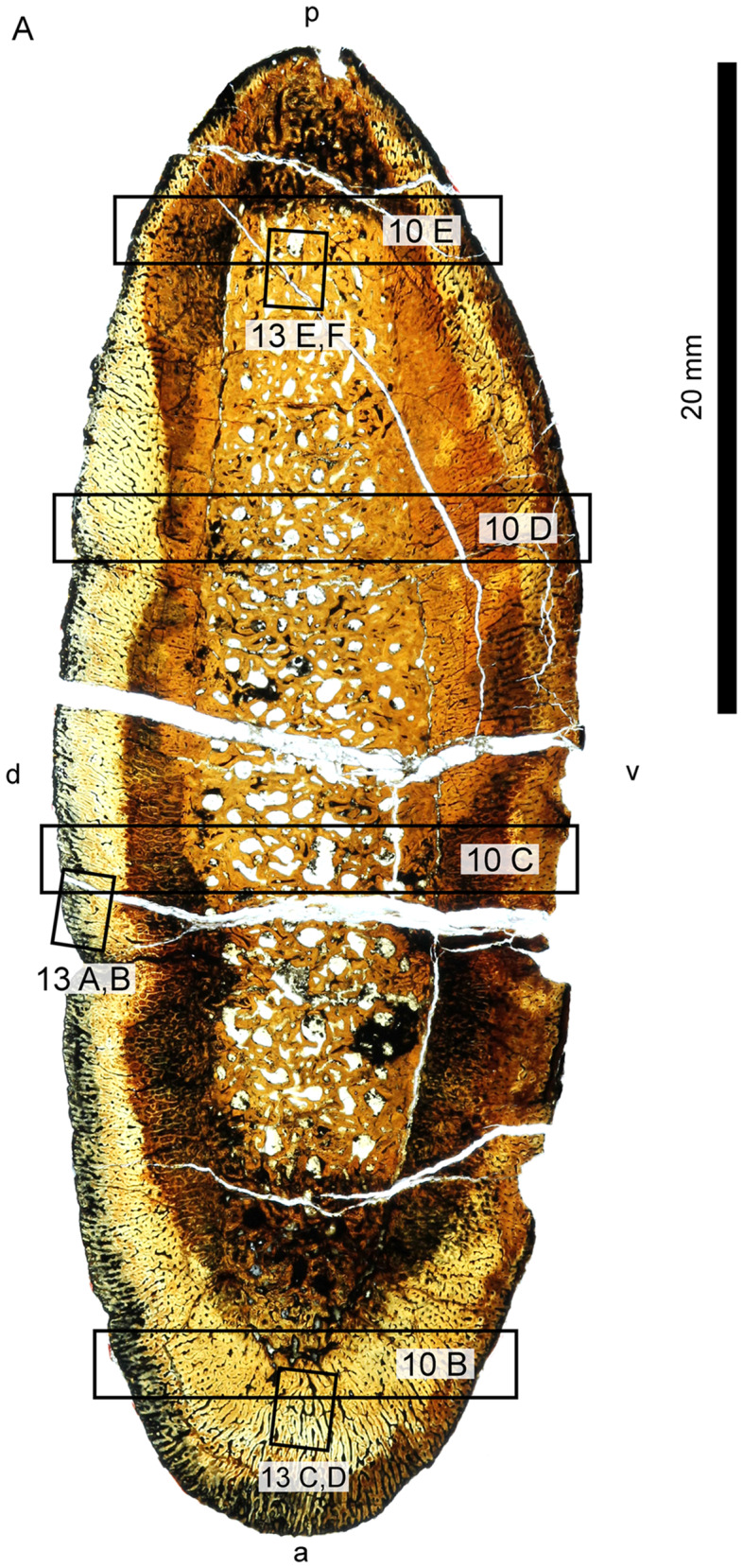

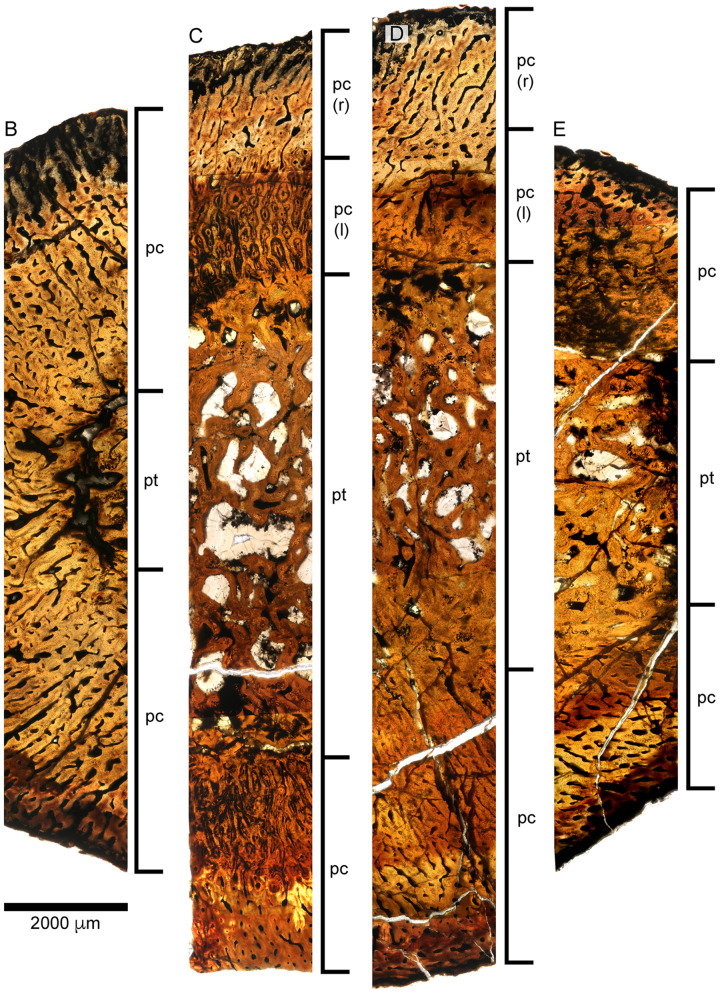
Overview (**A**) and microanatomy (**B**–**E**) of the fetal humerus, FMNH PR 1619. Black boxes indicate magnified regions in this figure and Fig. 13. a, anterior; d, dorsal; p, posterior; pc, primary cortex; pc (r), primary cortex (radial); pc (l), primary cortex (longitudinal); pt, primary trabeculae; v, ventral.

The small isolated humerus is depicted in Figs. 10 and 13. The cortex is thin relative to the endochondrum when compared to both *D. bonneri* specimens. Because all three elements are humeri and the sections were taken at similar locations, we believe this difference in cortical thickness is at least partially ontogenetic. The adult section is more distal and the cortex therefore thinner, so quantitative comparisons are impossible. The surface of the cortex is dark around its perimeter. Under high magnification the tissue here is degraded, and the feature is probably due to postmortem decay followed by infilling of dark sediment. The section is also stained dark to varying degrees, probably by kerogen derived from the surrounding shale. The cortex is highly vascularized. Near the endosteum this vascularity is longitudinal, but transitions through reticular to radial near the bone surface. The cortex comprises entirely primary woven fibered bone. The division of the cortex from the endochondrum is clear, with the heavily vascularized medullary region consisting of spongy trabeculae lined with primary lamellar bone, although some secondary lamellar bone is present. Islands of calcified cartilage are interspersed among the trabeculae; these are indicated by islands of large, globular chondrocytes surrounded by primary lamellar bone.

**Fig. 11:**
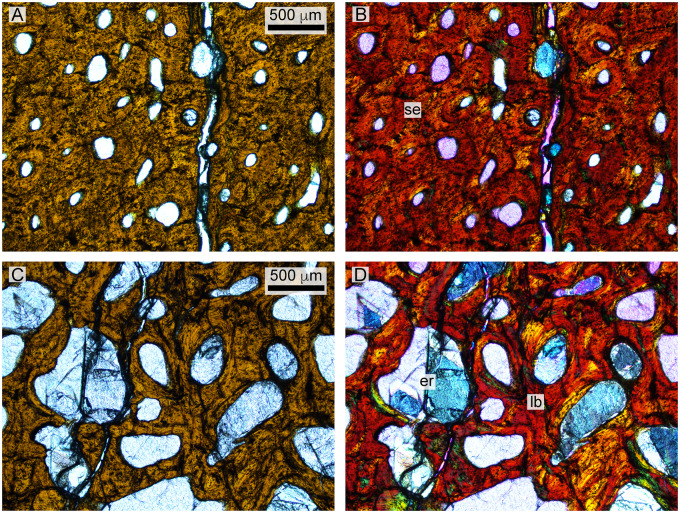
Histology of the *D. bonneri* adult. (**A** and **B**) show normal and polarized light views of the mid-cortex, consisting of secondary osteons. (**C** and **D**) show the trabecular region, consisting of secondary lamellar bone interspersed with erosion rooms. er, erosion room; lb, lamellar bone; se, seconday osteons.

**Fig. 12:**
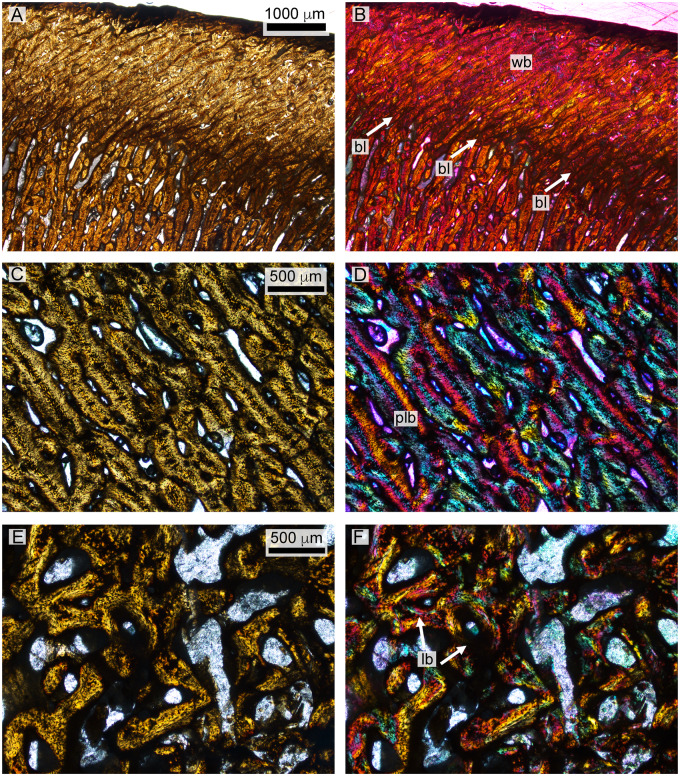
Histology of the *D. bonneri* neonate. (**A** and **B)** show the superficial cortex in the upper right quadrant, depicting the birth line in normal and polarized light. (**C** and **D)** show the mid-cortical region from the bottom right quadrant in normal and polarized light, consisting of quickly growing radially vascularized fibro-lamellar bone. Panels E and F show vascular cannals and primary lamellar bone from the medullary region in normal and polarized light with lambda filter. bl, birth line; lb, lamellar bone; plb, pseudolamellar bone; wb, woven bone.

**Fig. 13:**
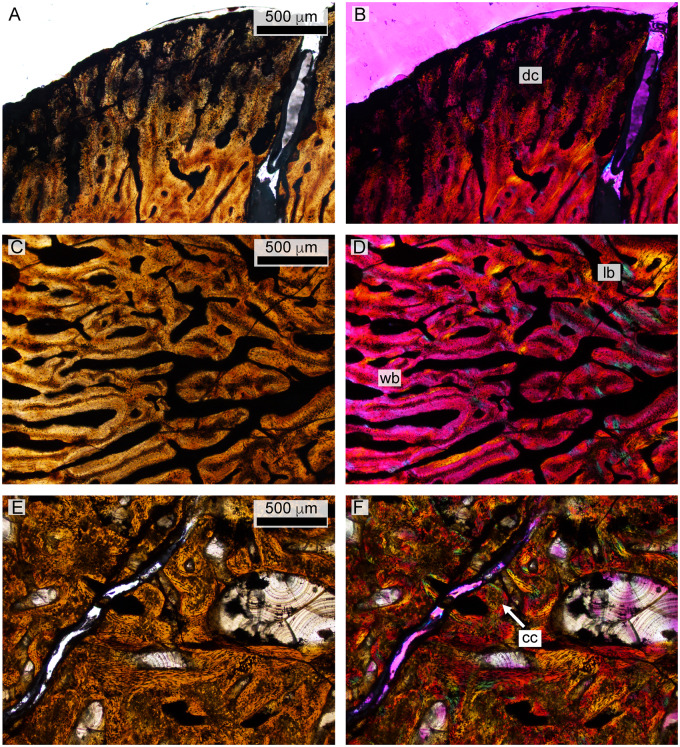
Histology of the polycotylid fetus. (**A** and **B)** depict the outer edge of the cortex, consisting of poorly ossified woven bone that may be partially decomposed. (**C** and **D**) show primary woven bone from the cortex in normal and polarized light with lambda filter. (**E** and **F**) illustrate medullary trabeculae composed of primary lamellar bone interspersed with calcified cartilage in normal and polarized light. cc, calcified cartilage; dc, decomposed woven bone; lb, lamellar bone; wb, woven bone.

**Fig. 14:**
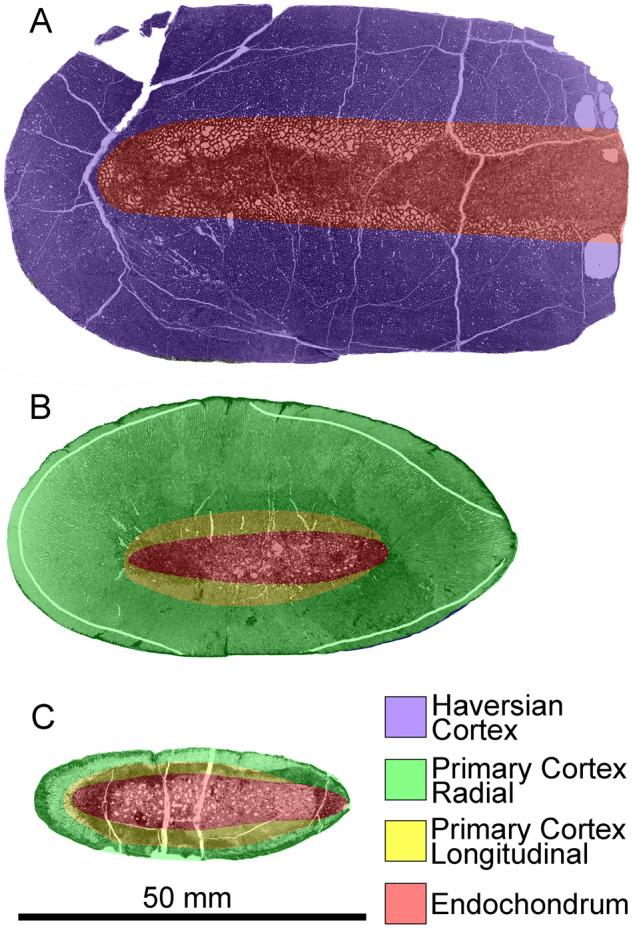
Interpreted histological sections of the *D. bonneri* growth series, adult UNSM 50133 (**A**); neonate UNSM 55810 (**B**); fetus FMNH PR 1619 (**C**). Shading demarcates regions of distinct vascular orientation; the neonatal birth line is shown in white in B.

## Discussion

Our discussion of the results presented above will begin with inferences concerning the *Polycotylus* mother and fetus. We then make ontogenetic assignments of the *D. osborni* material based on the findings from *Polycotylus*. Lastly, we attempt to integrate the observed morphology, microanatomy, and histology into a set of inferences concerning polycotylid life history.

### Adult polycotylid histology

The large LACM *Polycotylus* skeleton has fused neurocentral sutures in the vertebral column, indicative of skeletal maturity. The propodials are also adult in morphology, possessing large overall size, full ossification to both epiphyses, and sharp definition of the epipodial facets and groove between capitulum and tuberosity. All of these features are thought to indicate full adulthood in plesiosaurs (Brown 1981). The microanatomy and histology are also fully adult, identical to that seen in adult elasmosaurs (Wiffen et al. 1995), as well as adult true pliosaurs (Wintrich et al. 2017). The cortex is largely remodeled by longitudinal Haversian systems with the remodeling front having nearly reached the bone surface (Mitchell and Sander 2014, Mitchell et al. 2017), while the medullary region is filled with secondary trabeculae. As described above, a possible EFS is present in the adult *Polycotylus*, which in other amniotes indicates that skeletal maturity had been reached.

The *D. bonneri* adult does not have an EFS, but this is the only way it differs from the *Polycotylus* mother. Above we hypothesized that the large *D. bonneri* skeleton was an adult based on anatomical criteria; the *D. bonneri* propodials are a close match for that of adult *Polycotylus* morphologically, and the skeleton is bigger than the LACM mother. The histology of the large *D. bonneri* propodial is also identical to that seen in the mother *Polycotylus*, so if the *D. bonneri* was not a full adult it was close to fully grown. The lack of an EFS in the adult *D. bonneri* material may be due to weathering, or slightly larger adult body size in this taxon.

The erosion rooms enclosed by the spongy trabeculae in the medullary region seem to have been larger near the center in this humerus, accounting for the collapse and brittle deformation of the bone in this region. The much denser cortex is undeformed above and below the medulla, but heavily fractured at the leading edge. Given the level of remodeling there is no growth record preserved in the cortex of either adult humerus, although the secondary vascularization is oriented perpendicular to that of younger animals (Wintrich et al. 2017; see below). The vascular reorientation and high bone density may be driven by biomechanics; longitudinal osteons are best able to withstand bending forces on long bones (de Margerie et al. 2004).

### Polycotylid Fetal Histology

The fetus found with the *Polycotylus* mother is very young morphologically, documented at length in O’Keefe and Chiappe (2011). The fetal scapula sectioned here is ossified so poorly that it was originally identified as a humerus by those authors. The radical morphologic change of the polycotylid scapula during ontogeny is described by O’Keefe and Byrd (2012) and is not directly relevant here. Fetal microanatomy and histology are immature, differing from the mother in possessing a prominent Kastschenko’s line dividing the endochondrum and cortex. The medulla is spongy and highly vascular, consisting of primary lamellar bone and lacking calcified cartilage. The cortex was clearly deposited quickly, as all bone tissue is woven and heavily vascularized, with initial longitudinal canals giving way superficially to plexiform, and finally radial, vascularization. Radial vascularization is typical of juvenile plesiosaurs (Wintrich et al. 2017), and the change in vascularization orientation near the endochondrum implies that growth was accelerating at the time the fetus died (De Margerie et al. 2004). The histology of the fetal scapula implies that the fast, radial growth zone found in polycotylid propodials commenced before birth in the uterus, rather than after birth.

Above we hypothesized that the FMNH humerus was fetal, based on its overall size and poor ossification. The microanatomy and histology of this element is identical to that seen in the fetal scapula, with a highly vascularized spongy medulla lined with primary laminae. It also contains zones of calcified cartilage, unlike the fetal scapula. The cortex is also identical to that seen in the fetal scapula, comprising woven bone deposited around vascular canals whose initial longitudinal orientation transitions through plexiform to radial as growth accelerates. Based on comparison with the histology of the fetal scapula, we conclude that the FMNH humerus is also from a fetus, and that the initially deposited, radially vascularized cortex is a prenatal tissue in polycotylids.

### Polycotylid neonatal histology

The smaller *D. bonneri* skeleton was hypothesized to be near-natal in age based on size. The morphology of the element is odd; the long extensions of the endosteum past the edges of the cortical cup are unusual and not widely reported in plesiosaurs. The cortex is also thick through the center of the shaft relative to both the adult and the neonate. Both features imply that bone growth is allometric. The neonate humerus shape implies that cortical growth is positively allometric through the center of the shaft, and that growth of the cortical edge lags behind the wave of ossification moving up the endochondrum. The periosteal cuffs were therefore growing faster in girth than in length.

The microanatomy and histology of the neonate agree with this allometric assessment. The medulla of the neonate humerus consists of vascular canals lined with avascular lamellar primary bone. However some replacement has begun. This tissue is more ontogenetically mature than that seen in the fetal humerus, but still primary. It is also identical in size to the medulla in the fetal humerus. The deep cortex is also identical to that of the fetus, with an initial longitudinal vascularization giving way through plexiform to radial. Radially vascularized, woven to fibrolamellar bone comprises the remainder of the cortex and is very thick. The cortex clearly began growing quickly, then accelerated to grow very rapidly. As documented above in the LACM fetus, this very fast, radial growth phase began *in utero*. It terminated at birth, leaving evidence for this in the outer cortex. We therefore conclude that the animal was a neonate, not much larger than its size at birth.

The most superficial feature of the neonatal cortex is the abrupt change in vascular angle, and an increase in bone density. The bone around the rim of the neonate humerus is still woven and radially vascularized, but it is more dense than the bone below it, and is punctuated by gaps. It is therefore not a true LAG showing a drop in bone deposition rate. We instead interpret it as a birth line, indicating that fast cortical growth continued after birth but was now influenced by new, probably biomechanical, forces. There are biomechanical tradeoffs to fast bone growth. As documented by De Margerie et al. (2004), radially vascularized bones grow rapidly, but the bone tissue is oriented poorly to resist stresses incurred in locomotion. The fact that the osteons in fully adult polycotylids run at 90° to the radial vascularization in juveniles implies that polycotylids also made a tradeoff here, exchanging rapid intrauterine growth for juvenile bone strength. The poor biomechanical properties of radially vascularized bone may account for the positive allometry in shaft growth, with a relatively thick midshaft necessary to withstand locomotor forces outside the womb. We interpret the change in vascular angle and increase in bone density above the birth line as an initial response to more severe biomechanical demands of swimming.

### Inferences for polycotylid life history

Morphologic, microanatomic, and histologic analyses of the humeri reported here paint a coherent picture of life history in polycotylids (Fig. 14). The adults were large, active predators, whose osteosclerotic propodials were highly adapted to the demands of underwater flight. They were viviparous and gave birth to large young, on the order of 40% maternal length. The intra-uterine growth necessary to produce a progeny of this size was extremely rapid, as indicated by the wide zone of radially vascularized, woven to fibrolamellar bone. Rapid growth appears to have continued after birth, and we hypothesize that the new biomechanical constraints imposed by swimming may have produced the clear growth mark in the cortex. The rapidly deposited intra-uterine cortex may have been biomechanically weak, resulting in positive allometry in cortex thickness in response. Neonate propodials therefore possess inflated midshafts relative to those of adults.

The degree to which large birth size and rapid intrauterine growth occurred outside of derived polycotylids is currently unknown. *Polycotylus* and *D. bonneri* are closely related taxa, and it is unclear how pervasive their growth pattern is amongst other members of the clade. The large zone of radial cortical vascularity noted above is identical to that seen in the first plesiosaur, *Rhaeticosaurus*, and documented in several others (Wintrich et al. 2017); however, a feature like the polycotylid birth line is not seen. Instead the rapid radial growth terminates at what has been interpreted as a first year LAG that is histologically quite different from the polycotylid birth line. The genera *Plesiosaurus* and *Cryptoclidus* are again different histologically (Wintrich et al. 2017). Attributing this variability to biological differences in growth, phylogeny, or biomechanics is currently not possible. Further elucidation of reproductive biology within subclades will be a fruitful area of further research utilizing growth series of plesiosaur propodials.
